# Management-specific outcome evaluation of pituitary apoplexy; conservative and surgical approach

**DOI:** 10.1007/s11102-026-01695-5

**Published:** 2026-05-29

**Authors:** M. C. Guijt, M. J.T. Verstegen, A. H. Zamanipoor Najafabadi, L. E.H. Bakker, I. C. Notting, I. C.M. Pelsma, W. R. van Furth, N. R. Biermasz, K. M.J.A. Claessen

**Affiliations:** 1 Department of Medicine, Division of Endocrinology, and Centre for Endocrine Tumors Leiden, Leiden University Medical Center, Leiden, the Netherlands; 2 University Neurosurgical Center Holland, Leiden University Medical Center, Haaglanden Medical Center and Haga Teaching Hospitals, Leiden and The Hague, the Netherlands; 3 Department of Ophthalmology, Leiden University Medical Center, Leiden, the Netherlands

**Keywords:** Pituitary apoplexy, Conservative treatment, Surgical treatment, Endoscopic transsphenoidal pituitary surgery, Clinical outcomes, Visual acuity, Visual field defects, Hypopituitarism, Clinical decision making

## Abstract

**Purpose:**

Pituitary apoplexy can be managed by early surgery or a conservative approach, if necessary, followed by later elective surgery. Choice of management is related to correct diagnosis, symptom severity at presentation and symptom development prior to contacting the pituitary center of excellence (PTCOE). Specific circumstances may dictate a specific choice, in others shared decision-making is key. This retrospective cohort study aimed to describe clinical decision-making and outcomes of pituitary apoplexy treatment.

**Methods:**

Outcome measures were factors relevant to treatment choice, visual acuity (VA), visual field defects (VFD), cranial nerve palsies, pituitary function, measured at three time points (baseline, 6 months after diagnosis, last follow-up).

**Results:**

We studied 98 patients with clinical apoplexy diagnosed between 2005 and 2021. After the first clinical evaluation, 45 patients underwent early surgery and 53 initial conservative management. Main indications for early surgery were lowered VA and/or severe VFD. Postoperatively, mean VA improved from 0.63 (SD = 0.36) to 1.03 (SD = 0.3). VFD improved in 72.2% of patients. During continued follow-up, 21 initially conservatively treated patients had later elective surgery. VFD improved in 85.7% of these patients. Mean follow-up was > 38 months for each group.

**Conclusion:**

The choice for surgery is mainly driven by ophthalmological symptoms, in which severity determines its timing. Although ophthalmological recovery rates are reasonable, (endocrine) outcomes of apoplexy are unfavorable, irrespective of trajectory. Prospective studies are needed to assess optimal (timing of) treatment, in particular in those patients without an obvious reason for early surgery, taking into account the heterogeneity and variable course of this condition.

## Introduction

Pituitary apoplexy is a clinical syndrome caused by infarction or hemorrhage of a pituitary adenoma, often presenting with severe headache, (sub)acute cranial nerve deficits and hypopituitarism [1]. In patients with a known adenoma, the reported prevalence of pituitary apoplexy ranges from 0.6% to 13.0%, but for more than 75% of patients diagnosed with apoplexy, apoplexy is the first presentation of an underlying pituitary adenoma [2]. Known risk factors for pituitary apoplexy are the use of anticoagulants, hypertension, pregnancy, traumatic brain injury, tumor size, endocrine treatment with GnRH analogues or other stimulatory hormones, and recent (cardiothoracic) surgery, whereas in 10–40% of patients no clear risk factor can be identified [3–5].

A recent meta-analysis and another recent review showed that 75–100% of patients with pituitary apoplexy had a headache (usually the first symptom), 37% had a decrease in visual acuity (VA), 35% had visual field defects (VFD), 47% had dysfunction of other cranial nerves (CN), and 58% had endocrine dysfunction at initial examination [6, 7]. A significant subset of patients also experiences concomitant symptoms including nausea, vomiting, fever, photophobia, loss of consciousness or other focal neurological deficits. Approximately 75% of patients has an acute or subacute presentation of symptoms, whereas 25% has milder or less acute symptoms, resulting in a heterogenous case mix [8, 9]. Another subset of patients has signs of apoplexy on imaging or pathology results without any clinical manifestation, however, the term apoplexy is usually reserved for cases with a clinical syndrome. These differences in presentation have obvious impact on management decision and on urgency of intervention. Recently, we investigated the diagnostic journey of patients with apoplexy in our tertiary referral center in great detail. The variable clinical presentation often results in initial misdiagnosis and a significant delay in referral to an expert center, mainly in patients with a ‘non-classical’ presentation. We proposed a classification strategy distinguishing between acute, subacute and non-acute apoplexy subtypes [9], since these subtypes clearly have a different initial presentation, urgency, in-hospital route and different treatment strategies.

Both surgical intervention and conservative management are accepted treatment options in patients with pituitary apoplexy. In case of surgical treatment, the pressure on the pituitary gland and optic apparatus is immediately relieved, and necrosis of the endocrine gland is possibly prevented, with improvement of visual acuity (VA), visual field defects (VFD) and headache as a primary surgical goal. However, no consensus has yet been reached on the appropriate timing of surgical intervention in patients with acute symptoms, ranging from within 24 h to several days after apoplexy diagnosis in current literature. Published clinical outcomes are highly variable, but early surgical intervention seems to yield better ophthalmological outcomes [10–12] as well as positive effects on headache [13]. The effects of early surgery on endocrine outcomes of pituitary apoplexy are still poorly investigated.

If conservative treatment is chosen, most of the patients are hospitalized for proper monitoring of headache, neurological and ophthalmologic status, and fluid and electrolyte balance. Hormonal replacement therapy is initiated in case of (suspected) hypopituitarism [14]. Surgery can also be performed at a later stage for persistent or progressive compressive symptoms.

To date, published long-term outcomes of conservative and surgical treatment in patients with pituitary apoplexy are scarce. Weak evidence suggests an individualized choice, but considers surgery only in case of acute and severe visual deficits [7, 14]. In current practice, both surgery and conservative management are considered in many cases, especially in patients with a subacute presentation, resulting in a large practice variation [14–16]. The choice for surgical intervention is mainly based on ophthalmologic evaluation and less on patient’s endocrine function, while the latter also significantly impacts health-related quality of life (HRQoL) [14].

The present cohort study aimed at evaluating the decision-making process and key decision points in the choice for different treatment strategies for pituitary apoplexy. It describes the ophthalmological and endocrine outcomes across different subgroups (early surgery, continued conservative management, and elective surgery), accounting for differences in case mix.

## Methods

### Study design and patient selection

#### Study design

This is a single-center retrospective chart study with consecutive patients. We extracted data on the course of clinical symptoms over time, outcomes, and the clinical decision-making process from patient records at Leiden University Medical Centre (LUMC), which is a specialized tertiary referral center for pituitary care (Pituitary Tumor Center of Excellence, PTCOE). Patients were selected from existing patient registries and validated throughout our radiological information system. Approval for this study was granted by the institutional review board of the LUMC.

#### Patient selection

Records of 780 consecutive patients were screened who visited the department between 2005 and 2021, of whom 709 were identified from a LUMC database including all operated patients for a pituitary adenoma (from 2011 onwards, the electronic hospital system was introduced), and 71 additional patients were identified by an automated search through the radiology reports between 2005 and 2021. Inclusion criteria were: [1] symptomatic presentation, [2] radiologic signs suggestive of pituitary apoplexy on magnetic resonance imaging (MRI) or computer tomography (CT) scan, [3] sufficient data available for ophthalmologic and endocrine outcome evaluation, and [4] a minimal follow-up duration of 6 months after apoplexy diagnosis. We identified a total of eligible 103 patients, of whom five patients were excluded due to insufficient data availability, resulting in inclusion of 98 patients in the Leiden Apoplexy Cohort.

#### Treatment protocol

**Fig. 1:**
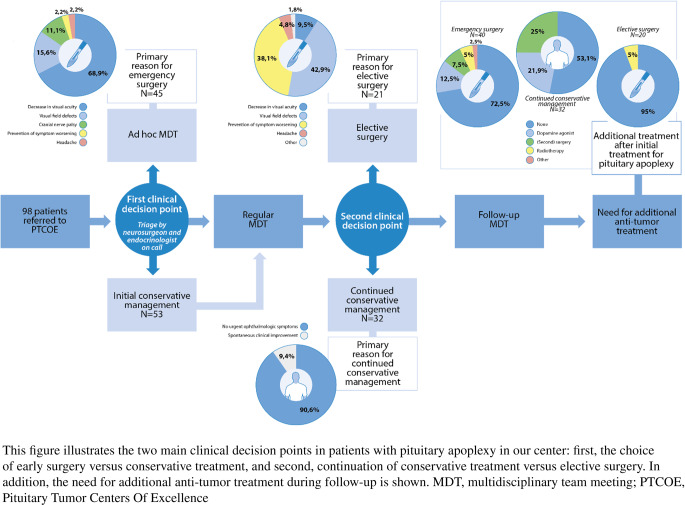
Treatment overview for pituitary apoplexy patients and main clinical reasoning for treatment choices

Patients suspected of pituitary apoplexy were referred to the LUMC either by their general practitioner or clinicians from referring hospitals. Depending on symptom severity, patients were seen at the emergency department, the ophthalmology department, or the combined outpatient clinic of the neurosurgeon and endocrinologist (with urgency or electively planned based on initial triage). Hospitalized patients from referring hospitals were admitted to our neurosurgical ward. All patients were discussed in the pituitary multi-disciplinary team (MDT) meeting to determine the best individualized treatment option, guided by the clinical presentation, comorbidity, and patient’s preference. The MDT included specialized neurosurgeons, endocrinologists, neuroradiologists and ophthalmologists, as well as specialized nurses in pituitary care (case managers). Individual treatment objectives, chances of achieving the treatment goals, the need for intervention and its optimal timing as well as suitable alternative treatment options were carefully considered and discussed with the patient. In case of a possible indication for early surgery, an ad hoc MDT with the (pituitary) specialists on call was organized to minimize delay in the decision whether to operate or not. In this respect, apoplexy patients ‘bypassed’ the regular preoperative care path of pituitary patients (See Fig. 1).

In case of conservative treatment, patients were admitted to the endocrinology ward or intensively monitored at the outpatient clinic, depending on symptom severity and presence of electrolyte disturbances. These conservatively managed patients received stress-dose hydrocortisone via continuous infusion, other hormonal supplementation and fluid resuscitation when indicated. Patients were closely monitored by endocrinologists and ophthalmologists with stringent follow-up of both pituitary and visual function. Regular reassessments, adjusted to individual cases, were performed to evaluate whether the conservative management could be continued safely or whether there were reasons to consider elective surgery due to apoplexy symptoms (i.e. persistent or progression of VFD, deterioration of VA, ongoing headaches).

In cases of (early) surgery, patients underwent operative intervention in addition to standard care (i.e. stress‑dose hydrocortisone via continuous infusion or dexamethasone in selected cases, other hormonal supplementation, and/or fluid resuscitation when indicated). Surgery was performed by two experienced pituitary surgeons using an endoscopic endonasal transsphenoidal approach [17]. Primarily, an attempt was made to achieve a total resection, balancing between total adenoma removal and optimal patient outcome. Then, variable consistency of adenoma tissue with elements of hemorrhage and/or infarction can complicate recognition of vital structures and may thereby lead to the perioperative decision to perform a subtotal resection or debulking with adequate decompression.

In the first year after apoplexy diagnosis, all patients were regularly monitored at the outpatient clinic and, on indication, re-evaluated in the MDT meeting. VA and VFD were evaluated within a week after the start of treatment, and every 3 months within the first year after surgery, or earlier in case of new symptoms. Pituitary function was assessed in the week after intervention, and generally at 6 weeks, 6 months, and a year after intervention, or more frequent when adjustments in hormonal supplementation were made. Follow-up MRI was usually performed 6 months after surgery, and earlier upon indication, and after 3 months in case of conservative management to assess for residual tumor, with a potential risk of a rebleed [18]. The postoperative care for patients with pituitary apoplexy was generally more intensive compared to non-apoplectic adenomas. Our case managers played an important role in this enhanced (postoperative) surveillance, including intensive coaching and education.

### Study parameters

#### Primary outcome measures

Primary outcome measures were the clinical decision-making process for treatment strategies in pituitary apoplexy patients (i.e. reasons for early surgery or initial conservative treatment, and later for continued conservative management or elective surgery), and ophthalmologic and endocrine outcomes over time.

##### Clinical decision-making process

The clinical decision-making on treatment choice was closely analyzed by reading MDT records and clinicians notes of outpatient clinical visits. Although the onset and course of symptoms in the period before entering our PTCOE clearly influenced treatment decisions, we decided not to include delay in referral, which was quite variable, in the definition of early vs. elective surgery. We defined early surgery as surgery performed within 72 h after referral to the LUMC, whereas elective surgery was defined as surgery after 72 h after entering our center in which the pituitary apoplexy was the decisive reason for surgery. This 3-day cut-off is based on previous retrospective studies [10–12].

##### Ophthalmological and endocrine outcomes

The following ophthalmologic and endocrine outcome measures were evaluated at the first presentation, 6 months after initial treatment and at last follow-up, and were described for the different treatment groups separately.

##### Ophthalmologic outcomes

Mean best corrected VA of both eyes (ODS), mean best corrected VA of the worst eye, VFD, and other cranial nerve (CN.) palsies CN. (III, IV or VI) were evaluated. VA was measured by experienced ophthalmologists using a Snellen chart [19]. VFD were evaluated using static perimetry by a Humphrey visual field analyzer [20, 21]. Over time, the course of VFD was defined as recovery, persistent loss or new loss, and was based on the ophthalmologist’s entry in the medical record.

##### Endocrine outcomes

Any form of hypopituitarism was assessed, stratified for insufficiency of individual hormone axes. Hypopituitarism was defined as clinically significant hormone deficiencies requiring hormone replacement of at least one pituitary axis, according to the latest guidelines [22, 23]. Over time, hypopituitarism was defined as never affected, recovered or ongoing loss (persistent loss combined with subsequent determined loss of pituitary function). It needs to be stated that in acute apoplexy presentation, the random sample assessing pituitary function is only partially representative to assess both under and over secretion. Even apparently normal values may not indicate preserved function.

#### Secondary outcome measures

Secondary outcome measures were: (1) additional anti-adenoma treatment during follow-up, i.e. (second) surgery (due to mass effect, adenoma growth or hormone overproduction), radiotherapy, or pharmacological treatment; and (2) complications of surgical treatment, including cerebrospinal fluid (CSF) leak, arginine vasopressin (AVP) deficiency, hemorrhage, infection, or syndrome of inappropriate antidiuretic hormone (SIADH).

#### Statistical analysis

For statistical analyses of the collected data, SPSS (version 25.0 [IBM Corp., Armonk, New York, USA]) was used. Descriptive data were reported as numbers (with percentages) and a mean or median, along with the standard deviation (SD) and range (minimum-maximum), respectively. Baseline characteristics and symptoms were compared between the subgroups at both decision‑making moments using either t‑test or chi‑square test, depending on the type of data. A t-test or McNemar’s test was used to compare numeric or categorical data between two time points within the same treatment group. For the analysis of the course of VA over time, a repeated measures ANOVA was conducted. *P* ≤ 0.01 was considered statistically significant.

It is important to emphasize that we have chosen not to compare the treatment strategies directly because the incomparability due to obvious case mix differences (baseline characteristics, clinical presentation) but to discuss the outcomes within each treatment subgroup separately.

## Results

### Clinical decision-making for treatment choice

Figure 1 addresses the main clinical decision-making points in the management of patients with pituitary apoplexy at first presentation in our center. The first main decision-making point comprises the decision for early surgery versus a conservative approach. Upon entering our PTCOE, 45 patients underwent early surgery (mean age 50.6, 64.4% male), whereas 53 patients were initially treated conservatively (mean age 45, 39.6% male). Notably, patients in the early surgery group were significantly more often male (p=0.01) compared with those in the initial conservative group. The primary reason for early surgery was impaired VA (69%), followed by severe VFD (16%), CN palsies other than of the optical nerve (11%) and severe headache (2%) in smaller subsets of patients. It is important to note that, although headache was not the primary indication in most patients who went for early surgery, our experience is that this frequently serves as a supporting factor in the decision for surgery. In the patients not operated in an early setting, due to milder presenting symptoms (see Table 1), a conservative treatment was initiated with close monitoring of electrolytes, pituitary function and ophthalmological situation.

The second main decision-making point is to assess whether the implemented conservative approach can be continued or whether surgery needs to be reconsidered. Of the 53 initially conservatively treated patients, 21 patients underwent elective surgery for pituitary apoplexy (mean age 49.9 years, 28.6% male) at a later stage after a median of 40 days (min-max: 3-130). The main reasons to choose elective surgery were persistent mild or progressive VFD (42.9%), prevention of further clinical (visual) deterioration (38.1%) and persistent headache (4.8%). In the other 32 patients, conservative management was continued (mean age 41.8 years, 46.9% male). Continuation of conservative treatment was mainly chosen in the absence of urgent visual symptoms (90.4%) or in case of spontaneous clinical improvement (9.6%). Surgical intervention was abstained from in one patient, as the diplopia was partly attributed to ophthalmologic comorbidity and there was a large delay in apoplexy diagnosis. In one patient with persisted mild CN palsy, surgery was performed in a later phase but primarily for hormonal overproduction rather than the apoplectic event itself. Mean follow-up duration since initial apoplexy presentation was 38.3 (SD = 32.2), 53.1 (SD = 41.1), and 46.0 (SD = 36.3) months for the patients who underwent early surgery, elective surgery and continued conservative treatment, respectively. All patient characteristics are presented in Table 1, stratified for the two main decision points.

**Table 1:** Baseline characteristics and clinical symptoms of pituitary apoplexy patients

Characteristics at baseline	First decision point			Second decision point		
	Early surgery *N* = 45	Initial conservative management *N* = 53	*p*-value	Elective surgery *N* = 21	Continued conservative management *N* = 32	*p*-value
Age at diagnosis, years (SD)	50.6 (16.2)	45.0 (16.7)	0.05	49.9 (15.1)	41.8 (17.2)	0.04
Male (N)	64.4% (29/45)	39.6%(21/53)	**0.01**	28.6% (6/21)	46.9% (15/32)	0.18
Mean BMI, kg/m² (SD)	28.0 (4.9)	28.7 (7.3)	0.31	29.0 (8.5)	28.5 (6.2)	0.41
Hypertension (N)	24.4% (11/45)	24.5% (13/53)	0.99	28.6% (6/21)	21.9% (7/32)	0.58
Diabetes mellitus (N)	20.0% (9/45)	1.9% (1/53)	**< 0.01**	4.8% (1/21)	0.0% (0/32)	0.21
Smoking (N)	11.4% (5/44)	13.2%(7/53)	0.78	9.5% (2/21)	15.6% (5/32)	0.52
Any ophthalmic comorbidity (N)	13.3% (6/45)	13.2% (7/53)	0.99	14.3% (3/21)	12.5% (4/32)	0.85
Anticoagulant use (N)	13.3% (6/45)	17.0% (9/53)	0.62	23.8% (5/21)	12.5% (4/32)	0.28
GnRH-agonist use (N)	6.7% (3/45)	1.9% (1/53)	0.23	0.0% (0/21)	3.1% (1/32)	0.41
Dopamine-agonist use (N)	6.7% (3/45)	15.1% (8/53)	0.19	9.5% (2/21)	18.8% (6/32)	0.36
Known adenoma prior to apoplexy (N)	35.6% (16/45)	30.2% (16/53)	0.57	28.6% (6/21)	31.3% (10/32)	0.84
*Size of adenoma*						
Micro adenoma (N)	0.0% (0/43)	15.1% (8/52)	0.027	9.5% (2/21)	19.4% (6/31)	0.28
Macro adenoma (N)	95.3% (41/43)	79.2% (42/53)		90.5% (19/21)	74.2% (23/31)	
Giant adenoma (N)	4.7% (2/43)	3.8% (2/53)		0.0% (0/21)	6.5% (2/31)	
Type of pituitary lesion*						
NFA (N)	73.3% (33/45)	54.7% (29/53)	0.06	52.4% (11/21)	56.3% (18/32)	0.24
Prolactinoma (N)	13.3% (6/45)	24.5% (13/53)		14.3% (3/21)	31.3% (10/32)	
RCC (N)	0.0% (0/45)	13.2% (7/53)		23.8% (5/21)	6.3% (2/32)	
Cushing (N)	4.4% (2/45)	1.9% (1/53)		4.8% (1/21)	0.0% (0/32)	
Acromegaly (N)	2.2% (1/45)	1.9% (1/53)		0.0% (0/21)	3.1% (1/32)	
Sheehan (N)	4.4% (2/45)	0% (0/53)		0.0% (0/21)	0.0% (0/32)	
Other (N)	2.2% (1/45)	3.8% (2/53)		4.8% (1/21)	3.1% (1/32)	
Visual acuity ODS (SD)	0.63 (0.36)	1.0 (0.22)	**< 0.01**	0.96 (0.26)	1.04 (0.20)	0.09
Visual acuity worst eye (SD)	0.50 (0.41)	0.96 (0.23)	**< 0.01**	0.91 (0.28)	0.99 (0.19)	0.09
VFD (N)	84.4% (38/45)	33.9% (18/53)	**< 0.01**	66.7% (14/21)	12.5% (4/32)	**< 0.01**
Headache (N)	97.7% (43/44)	84.9% (45/53)	0.03	85.7% (18/21)	84.4% (27/32)	0.89
Acute onset headache (N)	93.0% (40/43)	46.7% (21/45)	**< 0.01**	11.1% (2/18)	70.4% (19/27)	**< 0.01**
Other cranial nerve palsy (N)	71.1% (32/45)	18.9% (10/53)	**< 0.01**	19.0% (4/21)	18.8% (6/32)	0.98
Hypopituitarism of at least one axis (N)	77.8% (35/45)	67.9% (36/53)	0.28	66.7% (14/21)	68.8% (22/32)	0.87

Baseline characteristics and clinical symptoms of early surgery and initial conservative management patients, elective surgery and continued conservative management patients after initial conservative management

Data are presented as mean (SD) or % (N) unless stated otherwise. SD, standard deviation; N, number; GnRH, Gonadotropin-releasing hormone; BMI, body mass index; NFA, non-functioning adenoma; RCC, Rathke’s cleft cyst; ODS, left and right eye together; VFD, visual field defects

Differences in baseline characteristics and symptoms between the groups at both decision‑making moments were analyzed using either t‑tests or chi‑square tests, depending on the type of data

* Final clinical diagnosis of the pituitary lesion, based on (prior) hormonal assessment, radiology, and, when applicable, postoperative pathology results

### Clinical outcomes of different treatment strategies

#### Decision-making point 1: early surgery or initial conservative management

**Fig. 2:**
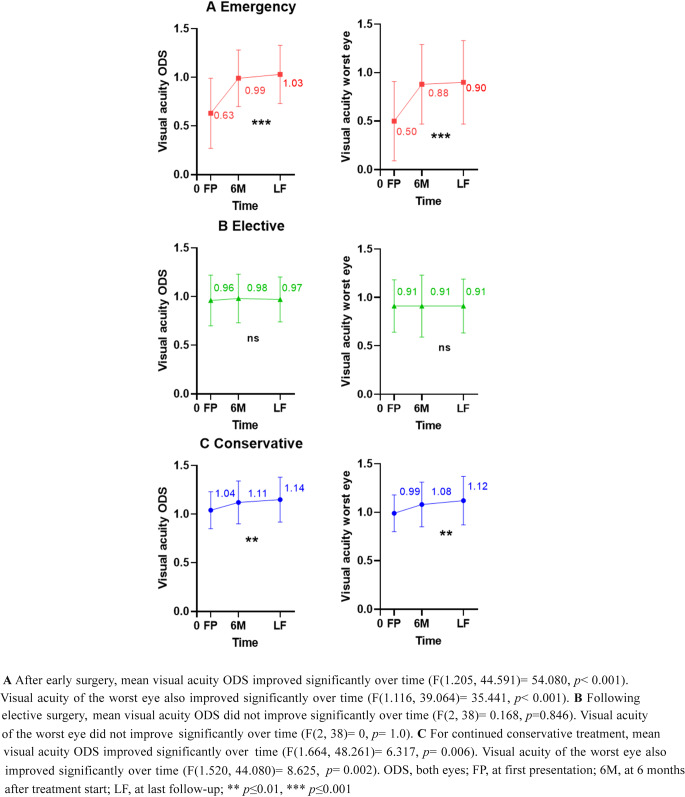
Course of mean visual acuity of both eyes and the worst eye over time after early surgery, elective surgery and continued conservative management

In the 45 patients that underwent early surgery, mean VA ODS showed a large improvement over time from 0.63 to 1.03 (*p* < 0.001; Fig. 2A). The largest improvement in VA was seen within the first 6 months after surgery (mean improvement 0.34 (0.27), *p* < 0.001), without further significant improvement afterwards. Moreover, VA of the worst eye improved significantly from 0.5 to 0.9 (*p* < 0.001; Fig. 2A). Upon early surgery, 72.2% of patients with VFD at baseline showed (complete) recovery from these symptoms (*p* < 0.001), whereas VFD persisted in 27.8% of patients. Recovery of other CN palsies was observed in 71.0% of patients, whereas 29.0% of patients had persistent symptoms after early surgery (*p* < 0.001).

Almost 80% of patients had objectified pituitary dysfunction of at least one hormonal axis at first presentation, mainly hypocortisolism (55.6%), hypothyroidism (64.4%) and hypogonadism (55.6%), with AVP deficiency in 4.4%. In the other 20% of patients, the hormonal situation at baseline was uncertain, with at least some measurable residual pituitary function without time for proper (dynamic) endocrine testing in the acute setting. After early surgery, recovery of pituitary function was seen in 15.2% of patients, with ongoing loss in 79.2% of patients. Of note, 9.5% of patients never had any hypopituitarism during follow-up. Details on the individual hormonal axes were shown in Table 2A.

**Table 2:** Pituitary dysfunction at first presentation, 6 months post apoplexy after treatment start and at last follow-up post apoplexy and several interventions

	Baseline		6 months			6 months	
2.A Early surgery (*N* = 45)	Loss of at least one axis (N)	Never affected ^a, d^	Recovered ^b, d^	Ongoing loss ^c, d^	Never affected ^a, d^	Recovered ^b, d^	Ongoing loss ^c, d^
Loss of at least one axis (N)	77.8% (35/45)	9.3% (4/43)	15.2% (5/33)	79.1% (34/43)	9.5% (4/42)	15.2% (5/33)	79.1% (34/43)
Hypocortisolism (N)	55.6% (25/45)	25.6% (11/43)	30.4% (7/23)	58.1% (25/43)	21.4% (9/42)	20.8% (5/24)	67.4% (29/43)
Hypothyroidism (N)	64.4% (29/45)	20.9% (9/43)	22.2% (6/27)	65.1% (28/43)	21.4% (9/42)	22.2% (6/27)	65.1% (28/43)
Hypogonadism (N)	55.6% (25/45)	20.9% (9/43)	16.7% (4/24)	69.8% (30/43)	21.4% (9/42)	17.4% (4/23)	69.8% (30/43)
Hyposomatotropism (N)	37.8% (17/45)	51.1% (22/43)	18.8% (3/16)	41.9% (18/43)	45.2% (19/42)	18.8% (3/16)	46.5% (20/43)
AVP deficiency (N)	4.4% (2/45)	83.7% (36/43)	0.0% (0/2)	16.3% (7/43)	81.0% (34/42)	0.0% (0/2)	16.3% (7/43)
2.B Elective surgery (*N* = 21)							
Loss of at least one axis (N)	66.7% (14/21)	28.6% (6/21)	7.1% (1/14)	66.7% (14/21)	28.6% (6/21)	14.3% (2/14)	57.1% (12/21)
Hypocortisolism (N)	28.6% (6/21)	66.7% (14/21)	16.7% (1/6)	28.6% (6/21)	66.7% (14/21)	16.7% (1/6)	23.8% (5/21)
Hypothyroidism (N)	57.1% (12/21)	33.3% (7/21)	16.7% (2/12)	57.1% (12/21)	28.6% (6/21)	16.7% (2/12)	61.9% (13/21)
Hypogonadism (N)	42.9% (9/21)	52.4% (11/21)	11.1% (1/9)	42.9% (9/21)	52.4% (11/21)	11.1% (1/9)	42.9% (9/21)
Hyposomatotropism (N)	23.8% (5/21)	76.2% (16/21)	0.0% (0/5)	23.8% (5/21)	76.2% (16/21)	0.0% (0/5)	23.8% (5/21)
AVP deficiency (N)	4.8% (1/21)	80.0% (16/20)	0.0% (0/1)	20.0% (4/20)	80.0% (16/20)	0.0% (0/1)	14.3% (3/21)
2.C Continued conservative management (*N* = 32)							
Loss of at least one axis (N)	68.8% (22/32)	32.3% (10/31)	4.8% (1/21)	64.5% (20/31)	29.0% (9/31)	22.7% (5/22)	56.3% (18/32)
Hypocortisolism (N)	53.1% (17/32)	48.4% (15/31)	31.3% (5/16)	35.5% (11/31)	48.4% (15/31)	47.1% (8/17)*	28.1% (9/32)
Hypothyroidism (N)	46.9% (15/32)	54.8% (17/31)	7.1% (1/14)	41.9% (13/31)	54.8% (17/31)	20.0% (3/15)	37.5% (12/32)
Hypogonadism (N)	50.0% (16/32)	51.6% (16/31)	0.0% (0/15)	48.4% (15/31)	48.4% (15/31)	18.8% (3/16)	43.8% (14/32)
Hyposomatotropism (N)	18.8% (6/32)	74.2% (23/31)	16.7% (1/6)	22.6% (7/31)	**7**1.0% (22/31)	33.3% (2/6)	18.8% (6/32)
AVP deficiency (N)	6.3% (2/32)	93.5% (29/31)	50.0% (1/2)	3.2% (1/31)	90.3% (28/31)	50.0% (1/2)	6.3% (2/32)

Data are presented as % (N). N, number; * *p* ≤ 0.01, ** *p* ≤ 0.001 vs. baseline at first presentation; a, never affected pituitary axis function during follow-up after 6 months and at last follow-up; b, recovery of pituitary axis dysfunction after 6 months and at last follow-up in patients with pituitary axis dysfunction at first presentation; c, Persisted or subsequently determined loss of pituitary axis function after 6 months and at last follow-up. d, Please note that occasionally data from one or two patients may be missing at certain time points, leading to slight variations in the numbers reported in the table

#It is important to note that especially in case of early surgery, there often is ‘no time’ for proper preoperative assessment of pituitary function because of the severity of visual symptoms. Therefore, the pituitary failure is a combined result of the apoplectic event resulting in pituitary damage and surgery, rather than surgery alone

#### Decision-making point 2: elective surgery or continued conservative management after initial conservative management

In the remaining 53 patients, initial conservative management was started. However, in 21 patients it was chosen to perform elective surgery at a later stage (median 40 days after initial diagnosis (min-max: 3-130). Below, we describe the ophthalmological and endocrine outcomes for both subgroups.

#### Elective surgery

Following elective surgery, there was no significant change in mean VA ODS over time (Fig. 2B), neither in VA of the worst eye (Fig. 2B*)*, given that at baseline the average best-corrected VA was near normal. Post elective surgery, 85.7% of patients who had VFD at baseline showed recovery (*p* < 0.001; Table 3B). In addition, all 4 patients (100%) with other CN palsies at baseline recovered after surgery.

**Table 3:** VFD and other cranial nerve palsies at first presentation, 6 months after treatment start and at last follow-up, stratified for early surgery, elective surgery and continued conservative treatment

	Baseline		6 months		Last follow-up		
3.A Early surgery(*N* = 45)		Persistent loss ^c^	Recovery ^a, c^	New loss ^b, c^	Persistent loss ^c^	Recovery ^a, c^	New loss ^b, c^
VFD (N)	84.4% (38/45)	40.5% (15/37)**	59.5% (22/37)**	0.0% (6/6)	27.8% (10/36)**	72.2% (26/36)**	14.3% (1/7)
Other cranial nerve palsy (N)	71.1% (32/45)	50.0% (15/30)**	50.0% (15/30)**	0.0% (0/13)	29.0% (9/31)**	71.0% (22/31)**	0.0% (0/12)
3.B Elective surgery (*N* = 21)							
VFD (N)	66.7% (14/21)	14.3% (2/14)**	85.7% (12/14)**	0.0% (0/7)	14.3% (2/14)**	85.7% (12/14)**	0.0% (0/7)
Other cranial nerve palsy (N)	19.0% (4/21)	0.0% (0/4)	100% (4/4)	0.0% (0/17)	0.0% (0/4)	100% (4/4)	5.9% (1/17)
3.C Continued conservative management (*N* = 32)							
VFD (N)	12.5% (4/32)	50.0% (2/4)	50.0% (2/4)	3.7% (1/27)	25.0% (1/4)	75.0% (3/4)	7.1% (2/28)
Other cranial nerve palsy (N)	18.8% (6/32)	16.7% (1/6)	83.3% (5/6)	0.0% (0/25)	16.7% (1/6)	83.3% (5/6)	0.0% (0/26)

Data are presented as % (N). N, number; * *p* ≤ 0.01, ** *p* ≤ 0.001 vs. baseline at first presentation; a, recovery of symptoms after 6 months and at last follow-up in patients with symptoms at first presentation; b, worsening of symptoms after 6 months and at last follow-up in patients with no symptoms at first presentation; c, Please note that occasionally data from one or two patients may be missing at certain time points, leading to slight variations in the numbers reported in the table

With respect to endocrine dysfunction, 14 patients (66.7%) had deficiencies in at least one pituitary axis at first presentation, mostly hypothyroidism (57.1%) and hypogonadism (42.9%). After surgery in an elective setting, the majority of patients had ongoing loss (57.1%) with recovery in only a minority of patients (Table 2B). In the elective surgery group, pituitary function was never affected in 28.6% of patients.

#### Continued conservative management

Within the 32 patients in whom the conservative treatment was continued, VA was near normal at presentation. However, we still observed a small but significant improvement from 1.0 to 1.14 over time without ophthalmological intervention (*p* = 0.006; Fig. 2C); this was also the case for the VA of the worst eye (0.99 to 1.1, *p* = 0.002; Fig. 2C). One patient had persistent mild VFD and one other patient had a persistent CN palsy during follow-up (Table 3C). In these two patients, there was abstained from surgery for reasons mentioned earlier (see paragraph clinical decision-making for treatment choice). Regarding endocrine dysfunction, 22 patients (68.8%) showed function loss of at least one pituitary axis at first presentation, especially of the corticotrope, thyrotrope and gonadotroph axes (respectively 53.1%, 46.9% and 50.0%). Table 2C shows the natural course of pituitary dysfunction in the continued conservative management group, showing some recovery of hormonal insufficiency, mainly of the corticotropic axis, in a subset of patients. Pituitary function remained unaffected in 29.0% of patients.

#### Additional anti-tumor treatment during follow-up and surgical complication rates

Figure 1 shows the additional anti-tumor treatment required during follow-up. Twenty-five percent (N=8) of conservatively treated apoplexy patients needed surgery at a later stage for other indications than apoplexy-related symptoms, mostly for persistent complaints of the underlying adenoma. In the early and elective surgery groups, respectively 27.5% and 5.0% of the patients required additional treatment after first surgery, mostly for a symptomatic residual adenoma. In this respect, re-surgery was needed in 7.5% of the patients after an early procedure, versus none of the electively operated patients. 

The surgical complication risk for pituitary apoplexy was limited, both for early as elective surgery, with an uncomplicated procedure in 77.8% and 76.2% of patients, respectively (Fig. 1). In the early surgery group (*N* = 45), six patients (13.3%) had CSF leak with a need for re-surgery, one patient had a meningitis (2.2%), one had a transient AVP-D (2.2%), one had a SIADH with a need for readmission and one had other complications (i.e. atrial fibrillation and delirium). One of the patients with CSF leak also had a transient CN palsy (N. VI). In the elective surgery group (*N* = 21), one patient (4.8%) had liquor leakage with a need for re-surgery, one (4.8%) had a hemorrhage of the carotid artery leading to transient cerebral ischemia, one had a transient AVP-D (4.8%), and two had persistent AVP-D (9.5%). One of the two patients with persistent AVP-D also had a nasal bleeding requiring surgical intervention.

## Discussion

In this large series of pituitary apoplexy patients in a tertiary referral center, we have evaluated outcomes of clinical pituitary apoplexy on two critical points in decision-making (Fig. 1). Previous studies [6, 15, 16, 24] have mainly compared outcomes between conservative and surgical management, which is, in our opinion, not feasible due to the heterogenous case mix and the marked different initial presentations of these patients. What is novel about our study is that outcomes are reported at key decision‑making moments instead, describing each subgroup separately and detailing the rationale behind treatment choices. This approach provides an overview of outcomes that is more closely aligned with real‑world clinical practice. In that clinical practice, patients with apoplexy are seen by the PTCOE after a variable period of delay. When a patient presents in the PTCOE, there is an initial triage to evaluate whether there is a possible indication for early intervention, being a first node in decision-making. Then, early surgical decompression is discussed, usually in an ad-hoc MDT. A second node is after the early phase, when ongoing headache, VFD progression, clinical deterioration or no signs of shrinkage may result in elective surgery.

### Decision-making around pituitary apoplexy treatment

A recent review by Biagetti et al. states that decision-making regarding the treatment of pituitary apoplexy is complex and largely based on expert opinion [7]. We identified two important decision-making points, the first being the choice to perform early surgery or not, and second whether conservative treatment can be continued, or elective surgery should be considered.

We demonstrated that in our center the choice for early surgery was primarily based on the ophthalmological status of the patient (i.e., decrease in visual acuity and/or VFD) at hospital entry, which is in line with the UK guideline and recent review by Biagetti et al. [7, 14]. The presenting endocrinological status and CN palsies (other dan VFD) of patients were not an important factor regarding the decision for early surgery. The presence of invalidating headaches was a supporting argument for surgery in most patients rather than the primary reason for early surgery. Several studies [25–27] have shown that men have a higher risk of pituitary apoplexy than women. Our study demonstrates that, at the initial decision‑making moment, early surgery is more frequently performed in men than for women. The exact reason for this difference remains unclear. This novel insight constitutes an interesting topic for future research.

The main reasons for elective surgery after initial conservative management in our study were progressive ophthalmological deterioration (worsening VFD or a decline in VA), ongoing headaches or the suspicion that persistent abnormalities might reasonably deteriorate further in the absence of surgical intervention.

### Ophthalmological outcome

Acute visual complaints, particularly a decrease in VA, are a key factor for clinical decision-making in patients with pituitary apoplexy. Most centers tend to operate patients with clinically relevant visual impairment soon after first presentation, which is confirmed in the present study. We found that early surgery (within 3 days after referral to our PTCOE) provided good recovery of VA an VFD, which is in line with available literature [6, 7, 10–12]. Recovery of VA occurred within the first 6 months after early intervention, not thereafter. In the other two groups (elective surgery and continued conservative treatment), VA was, on average, near normal. Interestingly, within this normal range of VA, we observed a small but statistically significant improvement over time, probably reflecting a certain relief of mass effect. Both patients operated in an early and elective setting had fair recovery from VFD and other CN palsies after intervention, being in line with the findings of a meta-analysis of Goshtasbi et al. [6]. Also in the continued conservative treatment group there was a clear trend toward VFD and other CN palsies improvement.

No patients in the conservative or elective surgery groups received high-dose corticosteroids. In the early surgery group, one patient received 10 mg perioperative dexamethasone for macroadenoma-related mass effect and subsequently developed severe psychosis. Two others received a single 4 mg perioperative dose, not considered high-dose. Prospective studies are needed to assess whether high-dose steroids improve outcomes in pituitary apoplexy.

Overall, we conclude that early surgery is beneficial for improving VA in pituitary apoplexy, whereas in the presence of minimal ophthalmological symptoms, conservative management is a reasonable option with close monitoring of ophthalmological parameters. If during follow-up VA deterioration or progressive VFD occur, elective surgery can subsequently be considered. If not or in the presence of CN palsies only, surgery does not necessarily lead to better outcomes, which was also described in the prospective study by Mamelak et al. [16].

### Endocrine outcome

At initial evaluation, presence of hypopituitarism was high in our cohort, with objectified pituitary dysfunction varying from 67% to 78% between the different treatment subgroups. Highest prevalence of pituitary dysfunction was seen in patients with the most severe apoplexy presentation requiring early surgery (up to 80%), largely comprising patients with macroadenomas or even giant adenomas.

In all treatment groups, hypocortisolism, hypothyroidism and hypogonadism were observed most frequently, but also prevalence of AVP-deficiency seemed to be slightly higher compared to non-apoplectic pituitary adenomas. The meta-analysis of Goshtasbi et al. showed endocrine dysfunction in about 58% of apoplexy patients at baseline [6], and also the recent prospective study of Mamelak et al., a retrospective study of Xiaoxu Li et al. and review of Biagetti et al. showed comparable numbers of pituitary disfunction [7, 16, 28].

Despite given treatment, ongoing hypopituitarism is frequent (ranging from 56% to 79% among treatment subgroups), with recovery of pituitary dysfunction in 15% to 23% of patients, being in line with previous studies [16, 24]. The high prevalence of ongoing hypopituitarism, irrespective of treatment modality, suggests irreversible damage to the pituitary gland. One of the difficulties in the interpretation of endocrine function is that, especially in acute apoplexy, the preoperative/baseline endocrine situation is uncertain in many patients, with at least some residual hormonal function at initial testing. In addition, it can be challenging to distinguish between, for example, central hypothyroidism and non-thyroidal illness in the acute setting. Finally, an AVP-deficiency can be unmasked by adequate steroid replacement and may be wrongly attributed to surgery when diagnosed in the postoperative phase. Taken all together, we interpret the pituitary dysfunction diagnosed postoperatively being most likely the consequence of the apoplexy event itself instead of a direct surgical effect, however, proper distinguishment between those two components is not possible. Furthermore, it is important to acknowledge that, in light of the severe visual symptoms, endocrine status is not a deciding factor for surgery.

Overall, endocrine outcome is rather poor in all treatment groups. There remains an unmet need to identify patients at risk of permanent pituitary dysfunction and to determine its predictors. In this regard, investigating baseline prolactin as a predictor of permanent hormonal deficiency may provide further insight [29].

### Strengths and limitations

Strengths of this study are the large number of analyzed apoplexy patients with limited missing data and the structured multidisciplinary approach. Another strength is the description of management-specific results, since outcomes are highly influenced by differences in initial presentation. Both patient and referral delay have a significant impact on treatment choice and outcome; this is especially true for the decision to perform early surgery or not. As a result, patient undergoing different treatment trajectories are essentially distinct patient groups, prohibiting direct comparison.

Limitations are the retrospective nature of this study, which prohibits proper analysis of patient-reported outcomes. In addition, as of 2015, less conservative and more strategic surgery was performed in patients with pituitary apoplexy in our tertiary center than before. An important factor in this respect is that, in our center’s experience, early surgical intervention appears to have a favorable impact on the debilitating headache symptoms. However, due to the retrospective nature of our study and lack of data on this topic, we were unable to reliably investigate this. Another limitation is the difficulty to assess pituitary function in the acute phase; often there is no time for proper (dynamic) endocrine testing.

## Conclusions

In this large series, we aimed to report overall and management-specific outcomes of patients with pituitary apoplexy, evaluating two critical points in treatment decision-making. We reported good overall outcomes of ophthalmological outcomes, but endocrine outcome is poor compared to non-apoplexy pituitary patients, irrespective of the chosen management strategy. There is a clear unmet need to improve the endocrine outcomes and QoL. Since apoplexy has a heterogenous presentation but with severe (permanent) deficits, this condition requires multidisciplinary expert care. Future prospective, and ideally randomized, studies are needed to assess best treatment strategy and its timing, especially in those cases where early surgery is not obviously indicated.

## Data Availability

Data is available on request.
